# Pass-through of the Oakland, California, sugar-sweetened beverage tax in food stores two years post-implementation: A difference-in-differences study

**DOI:** 10.1371/journal.pone.0244884

**Published:** 2021-01-04

**Authors:** Julien Leider, Yu Li, Lisa M. Powell

**Affiliations:** 1 Institute for Health Research and Policy, University of Illinois Chicago, Chicago, Illinois, United States of America; 2 Division of Health Policy and Administration, School of Public Health, University of Illinois Chicago, Chicago, Illinois, United States of America; Pennington Biomedical Research Center, UNITED STATES

## Abstract

**Introduction:**

Taxes on sugar-sweetened beverages (SSBs) have gained support as a policy response to adverse health effects associated with SSB consumption. On July 1, 2017, Oakland, California, implemented a one-cent/ounce tax on SSBs with ≥25 calories/12 fluid ounces. This study estimated the long-term impact of the tax on taxed and untaxed beverage prices.

**Methods:**

Data on 5,830 taxed and 5,146 untaxed beverage prices were obtained from 99 stores in Oakland and 111 stores in Sacramento (comparison site), California, in late May-June 2017 and June 2019. Linear regression difference-in-differences models were computed with store and product fixed effects, with robust standard errors clustered on store, weighted based on volume sold by beverage sweetener status, type, and size.

**Results:**

Taxed beverage prices increased by 0.73 cents/ounce (95% CI = 0.47,1.00) on average in supermarkets and grocery stores in Oakland relative to Sacramento and 0.74 cents/ounce (95% CI = 0.39,1.09) in pharmacies, but did not change in convenience stores (-0.09 cents/ounce, 95% CI = -0.56,0.39). Untaxed beverage prices overall increased by 0.40 cents/ounce (95% CI = 0.05,0.75) in pharmacies but did not change in other store types. Prices of taxed individual-size soda specifically increased in all store types, by 0.91–2.39 cents/ounce (p<0.05), as did prices of untaxed individual-size soda in convenience stores (0.79 cents/ounce, 95% CI = 0.01,1.56) and pharmacies (1.66 cents/ounce, 95% CI = 0.09,3.23).

**Conclusions:**

Two years following SSB tax implementation, there was partial tax pass-through with differences by store type and by beverage type and size within store type.

## Introduction

Sugar-sweetened beverage (SSB) consumption is associated with increased body weight and independently associated with type 2 diabetes and cardiovascular disease [1–4]. Furthermore, in 2015–2016, 53% of adults age 20 and over and 65% of children ages 2–19 failed to meet the 2015–2020 Dietary Guidelines for Americans recommendation of limiting added sugar intake to less than 10% of daily calories [5, 6]. SSBs were the largest source of added sugars in both children’s and adults’ diet [5, 6].

SSB taxes are supported as a public health policy to reduce consumption [7, 8] and are currently implemented in seven U.S. cities with one of those cities also taxing artificially sweetened beverages (ASBs) [9, 10]. On July 1, 2017, Oakland, California, implemented a one-cent/ounce tax on SSBs with at least 25 calories per 12 fluid ounces (hereafter “ounces”), exempting milk and 100% juice [11]. Oakland is currently one of four cities in the San Francisco Bay Area to implement an SSB tax. The tax was imposed on distributors and the extent to which it was passed through to consumers in the form of higher prices in the longer run is important for understanding impacts on purchasing, consumption, and health outcomes.

A number of studies have examined tax pass-through internationally [12]. In the U.S., studies have found a wide range of pass-through estimates. Two early studies of the Berkeley, California, one-cent/ounce tax found partial pass-through of 43–47% [13, 14], while a later study found varying pass-through by store type ranging from no to full pass-through [15]. A study of the San Francisco, California, one-cent/ounce tax found full pass-through of exactly 100% [16]. A study of the Boulder, Colorado, two-cent/ounce tax found approximately 79% pass-through [17]. More than full pass-through of 114–119% was found in two studies of the Cook County, Illinois, one-cent/ounce tax on both SSBs and ASBs [18, 19]. Two studies of the Philadelphia, Pennsylvania, 1.5-cent/ounce tax, also on both SSBs and ASBs, found 93% [20] and 105% [21] pass-through using store audit data, while another study using scanner data found pass-through of 43% in supermarkets, 58% in mass merchandise stores, and 104% in pharmacies [22]. Results ranged from partial to almost full pass through from three studies of the Seattle, Washington, 1.75-cent/ounce tax, from 59% based on store scanner data [23] to 89–97% based on store audit data [24, 25]. Two previous studies have assessed pass-through of Oakland’s tax, with one finding partial pass-through of 61% [26] and the other finding nearly full pass-through of 92% [16]. Of the 15 U.S. pass-through studies conducted so far, seven examined pass-through at most 1–4 months post-tax, two examined pass-through up to 6–9 months post-tax, and six examined pass-through at approximately one year post-tax, with none examining pass-through beyond one year post-tax.

Differences in pass-through by beverage type and size have been found in a number of U.S. studies [13–17, 19, 21–24, 26]. Further, while some found no or a limited impact on untaxed beverage prices [13, 17, 19, 22, 23], others found price increases [16, 21, 24–26]. A number also found differences by store type [13, 15–17, 21, 22, 24, 26]. These differences have varied widely across studies; for instance, while some found higher pass-through in pharmacies [22, 26], others found pass-through that was lower than or similar to that in other store types [13, 15–17, 21]. This may be a function not only of differences across jurisdictions and time periods but also of sampling variability from small store samples in some studies, e.g., 30 or fewer stores in several Berkeley evaluations for that intervention site [13–15].

This study used store audit data collected pre-tax and two years post-tax to examine pass-through by store type and beverage type and size of the Oakland SSB tax to taxed beverage prices as well as changes in untaxed beverage prices. To the authors’ knowledge, this is the first study not just for Oakland but of any local U.S. sweetened beverage tax to examine long-term pass-through by store type two years post-tax implementation.

## Methods

### Study sample

This study employed a difference-in-differences (DID) design using Sacramento, California, as the comparison site. Sacramento was chosen based on population size, percent non-Hispanic black, percent Hispanic, median household income, percent below 125% of the poverty threshold, and percent voting Democratic in the 2016 presidential election, using Mahalanobis matching [27]. This study was approved by the Institutional Review Board of the University of Illinois Chicago.

Data were collected in Oakland and Sacramento at baseline pre-tax implementation (late May to June 2017) and at a two-year follow-up (June 2019). Details on sampling have been provided elsewhere [28]. Briefly, seven store types were sampled in 16 areas in each site based on random points drawn using ArcGIS 10.4: general merchandise stores, supermarkets, grocery stores, chain convenience stores, non-chain convenience stores, small discount stores, and drug stores/pharmacies (hereafter “pharmacies”). General merchandise stores were specifically defined as Walmart, Target, K-Mart, and Meijer. Stores that sold fresh meat were classified as supermarkets if they had two of three specified service counters (butcher, bakery, and deli) and as grocery stores otherwise. Stores that did not sell fresh meat were classified as small discount stores if they mentioned discounts or dollar store in their name, pharmacies if they sold prescription drugs, and as convenience stores otherwise. Stores were audited using the Beverage Tax Food Store Observation Form [29], which has been shown to have high inter-rater reliability [30]. Data were collected for 132 beverage products (e.g., 12 ounce Pepsi). Data were entered in 2017–2019 using Research Electronic Data Capture (REDCap) databases [31].

At baseline, data were collected from 129 stores in Oakland and 124 in Sacramento. At two-year follow-up, 106 of these stores in Oakland and 119 in Sacramento were audited (differences due to, e.g., store closures, auditors asked to leave or feeling unsafe). Only stores that could be audited at both time points were included in the analytical sample. Price observations were included only when obtained for a given product and store at both time points. General merchandise and small discount stores were excluded as there were no observations in Oakland for either after these restrictions. The final analytical sample included 5,830 taxed SSB price observations, 3,096 untaxed ASB price observations, and 2,050 untaxed unsweetened beverage (USB) price observations from 99 stores in Oakland and 111 stores in Sacramento, with sample sizes by store type shown in Table 1.

**Table 1:** Weighted mean price of taxed and untaxed beverages by store type, site, and time period.

	Oakland		Sacramento	
	Baseline	24 Months	Baseline	24 Months
**Supermarkets and Grocery Stores**				
Taxed beverages (*n* = 452, 452, 806, 806)	4.87	5.77	4.52	4.69
Soda (*n* = 195, 195, 357, 357)	3.57	4.53	3.28	3.47
Individual-size (*n* = 70, 70, 108, 108)	8.06	9.69	8.25	8.82
Untaxed beverages (*n* = 429, 429, 804, 804)	4.25	4.41	4.25	4.46
Soda (*n* = 124, 124, 256, 256)	4.35	4.59	3.75	3.85
Individual-size (n = 44, 44, 72, 72)	8.89	9.88	8.80	9.29
**Convenience Stores**				
Taxed beverages (*n* = 319, 319, 767, 767)	9.79	10.49	7.72	8.52
Soda (*n* = 147, 147, 333, 333)	5.84	6.71	4.32	4.71
Individual-size (*n* = 122, 122, 207, 207)	8.06	9.60	8.21	8.84
Untaxed beverages (*n* = 205, 205, 554, 554)	7.73	8.47	7.06	7.56
Soda (*n* = 71, 71, 181, 181)	7.52	8.75	5.95	6.49
Individual-size (*n* = 66, 66, 144, 144)	8.40	9.86	8.37	9.04
**Pharmacies**				
Taxed beverages (*n* = 219, 219, 352, 352)	5.29	6.35	5.12	5.44
Soda (*n* = 108, 108, 164, 164)	3.70	4.91	3.52	3.94
Individual-size (*n* = 32, 32, 39, 39)	8.15	10.97	8.44	8.88
Untaxed beverages (*n* = 230, 230, 351, 351)	4.67	5.25	5.17	5.36
Soda (*n* = 92, 92, 128, 128)	4.04	4.79	3.82	4.25
Individual-size (*n* = 30, 30, 35, 35)	8.24	10.36	8.40	8.87

Mean prices in cents per ounce are shown, weighted based on the distribution of volume sold by beverage sweetener status (sugar-sweetened, artificially sweetened, or unsweetened), type, and size in Oakland, Sacramento, and 2-mile border areas around both sites in June 2016 to May 2017, computed by the authors from Nielsen scanner data. Sample sizes by site and time period are shown in parentheses for each row. Sample sizes by store type in Oakland and Sacramento, respectively, are as follows: 17 and 21 supermarkets, 14 and 11 grocery stores, 22 and 38 chain convenience stores, 32 and 24 non-chain convenience stores, and 14 and 17 pharmacies.

### Measures

Beverages were classified as taxed and untaxed and by type. Taxed beverages included soda, sports drinks, energy drinks, juice drinks, and ready-to-drink tea and coffee (hereafter “tea/coffee”), and untaxed beverages included diet versions of taxed beverages and water, milk, and 100% juice. Beverages were classified as individual-size (single items ≤1 liter) or family-size.

The analytical price measure captured the price displayed on the shelf tag, and was computed as (1) the regular price for beverages that were not on sale or (2) the sale price for beverages that were on sale. Only reduced-price and reduced-price-per-quantity sales, where beverages are sold for a constant price per unit, were counted for this purpose. No purchases were made to verify the price at the register, but for selected products, data collectors were instructed to ask for the price if it was not displayed.

### Statistical analysis

Linear regression DID models were computed with store and product fixed effects, with robust standard errors clustered on store, using the *areg* command in Stata/SE, version 15.1. Specifically, models were computed of the form

$$Price_{ist}=\beta_{0}+\beta_{1}Posttax_{t}+\beta_{2}Posttax_{t}\cdot Oakland_{s}+\alpha_{i}+\gamma_{s}+\;\epsilon_{ist}$$ (1)

where *i* indexes products, *s* indexes stores, and *t* indexes time (pre-/post-tax), with *Posttax* being an indicator for the post-tax period and *Oakland* an indicator for observations from Oakland (as opposed to Sacramento). Analyses were weighted based on the distribution of volume sold by beverage sweetener status (SSB, ASB, or USB), type, and size in Oakland, Sacramento, and 2-mile border areas around both sites from June 2016 to May 2017, computed by the authors from Nielsen scanner data excluding sizes larger than those present in the audit data. Statistical significance was determined at a threshold of p<0.05. Models were estimated to examine changes in price for taxed and untaxed beverages overall. Additionally, estimates are presented from models for soda and individual-size soda to examine possible differences by beverage type and size, since the greatest number of observations by type was available for soda.

DID models rely on the assumption that price trends in Oakland and Sacramento would have been parallel in the absence of a tax. Because only one pre-tax time point is available, parallel trends in the pre-tax period (as a proxy for this assumption) cannot be tested with the data used in this study. Tests of parallel trends from August 2016 to May 2017 were conducted using universal product code-level Nielsen scanner data by testing the significance of interaction terms in linear regressions of price on month, an indicator for Oakland, and interaction terms between month and the Oakland indicator. These tests failed to reject parallel trends for taxed and untaxed beverages overall and by beverage type and size at a p<0.10 significance threshold.

## Results

Table 1 shows the weighted mean price of taxed and untaxed beverages by store type, site, and time period. Taxed beverage prices increased by 0.90–1.06 cents/ounce on average in Oakland in supermarkets/grocery stores and pharmacies, corresponding to roughly the full amount of the tax, while increasing by 0.17–0.32 cents/ounce on average in Sacramento. In convenience stores, prices increased by 0.70 cents/ounce on average in Oakland but increased by an even larger amount, 0.80 cents/ounce, in Sacramento. Untaxed beverage prices changed by relatively small amounts in both sites in supermarkets and grocery stores, while increasing somewhat in convenience stores and pharmacies (particularly in Oakland).

Table 2 shows the estimated impact of the Oakland SSB tax on prices in Oakland relative to Sacramento by store type from DID models (i.e., the β_2_ coefficient from the model as shown above). Results for the other non-fixed effect term in these models, showing estimated changes in Sacramento and corresponding to the β_1_ coefficient from the model, are shown in S1 Table. Overall, pass-through for taxed beverages was approximately three-quarters of the tax in supermarkets and grocery stores (0.73 cents/ounce, 95% CI = 0.47, 1.00) and pharmacies (0.74 cents/ounce, 95% CI = 0.39, 1.09). However, overall, there was no statistically significant pass-through in convenience stores and the point estimate was close to zero (-0.09 cents/ounce, 95% CI = -0.56, 0.39). Tests revealed statistically significant differences in pass-through between convenience stores and all other store types (where p<0.01 for all pairwise comparisons, based on interaction terms) but not between other store types. The pattern of pass-through was similar for taxed soda. As a sensitivity check, unweighted analyses were also conducted. The pattern of results was similar for taxed beverages overall, although point estimates were larger in some store types: 0.85 cents/ounce (95% CI = 0.54, 1.16) in supermarkets and grocery stores, -0.06 cents/ounce (95% CI = -0.60, 0.48) in convenience stores, and 1.45 cents/ounce (95% CI = 0.91, 1.99) in pharmacies.

**Table 2:** DID estimated impact of the Oakland, California, sugar-sweetened beverage tax on beverage prices, 2017–2019.

	Supermarkets and Grocery Stores	Convenience Stores	Pharmacies
	Coef. (95% CI)	Coef. (95% CI)	Coef. (95% CI)
Taxed beverages (*n* = 2516, 2172, 1142)	0.73 (0.47, 1.00)	-0.09 (-0.56,0.39)	0.74 (0.39,1.09)
Soda (*n* = 1104, 960, 544)	0.78 (0.48, 1.07)	0.48 (-0.18,1.15)	0.80 (0.41,1.18)
Untaxed beverages (*n* = 2466, 1518, 1162)	-0.06 (-0.21, 0.09)	0.26 (-0.19,0.70)	0.40 (0.05,0.75)
Soda (*n* = 760, 504, 440)	0.14 (-0.12, 0.41)	0.70 (-0.08,1.47)	0.33 (-0.30,0.95)

DID estimates comparing pre-post changes in prices in cents per ounce in Oakland, California, to those in Sacramento, California, from linear regression models with store and product fixed effects, with robust standard errors clustered on store, are shown. Models were weighted based on the distribution of volume sold by beverage sweetener status (sugar-sweetened, artificially sweetened, or unsweetened), type, and size in Oakland, Sacramento, and 2-mile border areas around both sites in June 2016 to May 2017, computed by the authors from Nielsen scanner data. Sample sizes by store type are shown in parentheses for each row.

Abbreviations: DID, difference-in-differences.

There was no statistically significant change in untaxed beverage prices overall relative to Sacramento for any store type except pharmacies (increase of 0.40 cents/ounce, 95% CI = 0.05, 0.75). There was no statistically significant change in the price of untaxed soda specifically in any store type.

**Fig 1:**
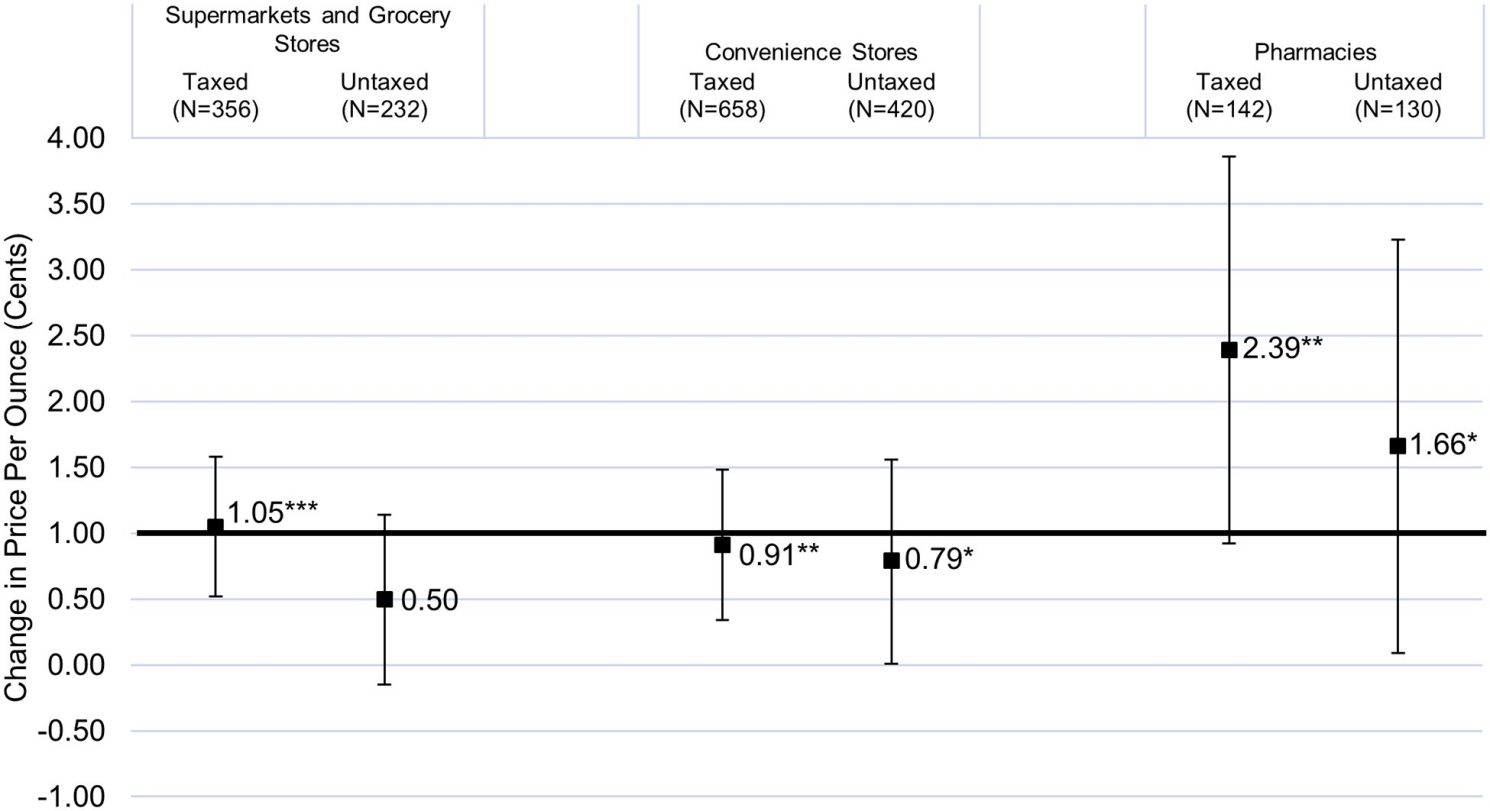
DID linear regression model estimates of pre-post change in prices (cents/ounce) of taxed and untaxed individual-size soda in Oakland, California, relative to Sacramento, California, 2017–2019. * p<0.05, ** p<0.01, *** p<0.001. Estimates are shown with 95% confidence intervals from models with store and product fixed effects, with robust standard errors clustered on store. Models were weighted based on the distribution of volume sold by beverage sweetener status (sugar-sweetened, artificially sweetened, or unsweetened), type, and size in Oakland, Sacramento, and 2-mile border areas around both sites in June 2016 to May 2017, computed by the authors from Nielsen scanner data. Abbreviations: DID, difference-in-differences.

Changes in price were further examined for individual-size taxed and untaxed soda. Fig 1 shows estimated price changes in Oakland relative to Sacramento for individual-size taxed and untaxed soda by store type from DID models. Results for the other non-fixed effect term in these models, showing estimated changes in Sacramento, are shown in S1 Table. Pass-through for individual-size taxed soda was almost complete, complete, or over-shifted in all store types: supermarkets and grocery stores (1.05 cents/ounce, 95% CI = 0.52, 1.58), convenience stores (0.91 cents/ounce, 95% CI = 0.34, 1.48), and pharmacies (2.39 cents/ounce, 95% CI = 0.92, 3.86). For individual-size untaxed soda, there was no statistically significant change in price relative to Sacramento in supermarkets and grocery stores; however, the increase was nearly as large as that for individual-size taxed soda in convenience stores (0.79 cents/ounce, 95% CI = 0.01, 1.56) and pharmacies (1.66 cents/ounce, 95% CI = 0.09, 3.23).

## Discussion

This study of pass-through of the Oakland SSB tax contributes to the literature by being the first to examine longer-term pass-through of any local U.S. sweetened beverage tax two years post-tax implementation. Several differences in tax pass-through were found both across and within store types. Pass-through for taxed beverages overall was 73–74% for most store types but was not found in convenience stores. However, within convenience stores, nearly full pass-through (91%) was found for taxed individual-size soda and, interestingly, a similar price increase was found for untaxed individual-size soda (0.79 cents/ounce). A similar pattern was found in pharmacies, with price increases of 2.39 cents/ounce for taxed individual-size soda and 1.66 cents/ounce for untaxed individual-size soda. That is, in these smaller store types, prices increased for smaller convenience sizes of soda regardless of whether or not they were taxable, perhaps reflecting relatively inelastic demand for these products and/or a flat pricing strategy that does not differentiate taxed and untaxed soda in order to make up for limited tax pass-through on taxed products overall. In contrast, in supermarkets and grocery stores, price increases were found for taxed but not untaxed individual-size soda. Since these findings are from two years post-tax implementation when stores had had time to adjust to the tax, they are unlikely to reflect short-run differences in implementation capacity by store type.

As reported earlier, tax pass-through results have varied widely across studies, including by store type. Compared with the two previous studies of Oakland’s SSB tax, which found 61% [26] and 92% [16] pass-through one year post-tax, our study estimated overall pass-through for taxed beverages at two years post-tax to be between these two estimates in supermarkets, grocery stores, and pharmacies (73–74%) while not being found in convenience stores. In terms of store type differences, one previous Oakland study found, relative to large grocery stores, higher pass-through for pharmacies but no differences for other store types [26], while the other found full pass-through to overshifting in chain convenience, corner, and liquor stores and differences across store types in exploratory analyses combining Oakland and San Francisco (another taxing jurisdiction) that were admittedly underpowered [16]. Our findings of no overall pass-through in convenience stores, but approximately three-quarters pass-through in all other store types, also contrast to study results from other U.S. jurisdictions. While one study of Berkeley’s tax found no pass-through in independent corner stores and gas stations but partial or full pass-through in other store types (including chain gas stations) [15], two other studies of Berkeley’s tax found no differences by store type [14] or equal or higher pass-through in convenience and liquor stores compared to other store types [13]. Further, a study of Boulder’s tax found higher pass-through in convenience and liquor stores compared to other store types [17], and a study of Philadelphia’s tax found the highest pass-through in gas stations [21]. Two studies of Seattle’s tax found similar pass-through for taxed beverages by store type, including small stores (which correspond to our convenience stores) [24, 25].

Noteworthy in our study is that while we 1) found no overall increase in taxed beverage prices in convenience stores, and 2) generally found no overall changes in untaxed beverage prices (except a moderate increase in pharmacies), we did find that the price of both taxed and untaxed individual-sized soda increased in convenience stores (by 0.91 and 0.79 cents/ounce, respectively), with a similar pattern for pharmacies. Regarding changes in prices of untaxed beverages, one previous study of Oakland’s tax found a marginally significant price increase of 21% of the tax, driven by an increase in the price of diet soda [26], while the other found price increases of 33–78% of the tax for specific sizes of diet soda (<33.8oz and >1.25L) but not overall [16]. However, neither study assessed beverage types by store type, so it is unclear if their findings for diet soda reflect our patterns by store type [16, 26]. Indeed, samples sizes in the previous Oakland studies were more limited; e.g., for the one study, its sample sizes for convenience stores (10 in Oakland and 15 in its comparison area) and gas stations (6 in Oakland and 8 in its comparison area) were substantially lower than our sample size for convenience stores (54 in Oakland and 62 in Sacramento) [26], while the other also had lower sample sizes for corner/small grocery stores (16 in Oakland and 31 in comparison cities), chain convenience stores (5 in Oakland and 8 in comparison cities), and liquor stores (4 in Oakland and 7 in comparison cities) relative to our sample size for convenience stores [16].

The results from this study suggest that store types that may be more likely to sell “convenience” purchases of SSBs increase prices of both regular and diet soda. Few previous studies examining changes in taxed and untaxed beverage prices by both beverage and store type are available for comparison. Different to our study, a study of Berkeley’s tax found higher prices for regular soda but not diet soda in supermarkets, small grocery, and convenience stores and no significant price changes for either in drugstores or liquor stores [13]. However, consistent with our study, a study of Seattle’s SSB tax found that in small store types, prices increased for both taxed and untaxed beverages overall, particularly for taxed and untaxed soda, whereas in larger store types (supermarkets and superstores), pass-through was found for taxed beverages but no change in price was found for untaxed beverages [24].

The results of our study showing differences in tax pass-through by store type suggest that there may be differential tax impacts on purchasing/consumption, as previous research has found disparities in food store access by income and race/ethnicity [32–35]. Lower-income neighborhoods tend to have lower access to supermarkets, as do predominantly black neighborhoods, making residents more reliant on smaller stores such as convenience stores. Our finding of no overall pass-through in convenience stores suggests the impact of the tax for lower-income and minority communities may be dampened. This is also true of our finding of similar pass-through in convenience stores and pharmacies for taxed and untaxed individual-size soda, which removes a potential incentive for consumers to reduce their intake of sugar by switching from SSBs to artificially sweetened beverages, although the incentive still remains to switch to other untaxed beverages.

### Limitations

This study is subject to several limitations. First, data were collected on a limited set of commonly sold products on the audit form. Second, several studies have found instances where a tax placed on distributors was added at the register by stores rather than being incorporated in the shelf price [16, 17, 24, 26]. We did not purchase beverages during store audits, so our price measure would only include the tax in such instances if data collectors happened to note a shelf tag indicating the tax would be added at the register. However, the previous Oakland studies both found this in fewer than ten stores [16, 26]. Third, given differences in tax pass-through by beverage type and size, weights such as those used in this study can play an important role in ensuring store audit data more accurately reflect the distribution of volume sold. However, we do not have data on the distribution of volume sold by store type, which is likely to vary, so we are unable to implement store type-specific weights. This may be particularly important for convenience stores where “convenience purchases” such as individual-size soda may be more prevalent. Fourth, while Sacramento was chosen as a comparison site based on matching on several important demographic and socioeconomic characteristics and exhibited similar price trends in the pre-tax period, we cannot rule out or control for the possibility of some interventions or changes in policies or programs in the post-tax period, which could affect the extent to which Sacramento serves as a good counterfactual. Fifth, the Stata *areg* command was used to compute models with store and product fixed effects under the assumption that the number of stores and products would be fixed if the sample size were to be increased. It would have been preferable to use the *xtreg* command, which assumes an increasing number of fixed effect units as the sample size increases, but *xtreg* does not permit the use of weights that vary within fixed effect units. However, as a sensitivity analysis, unweighted results for taxed and untaxed beverages overall in all three store types were compared from *areg* and *xtreg* assuming the number of stores would increase as the sample size increases, and the results were nearly identical. Finally, because there were only two sites, the standard errors (clustered on store) are likely underestimated [36], as has been acknowledged in other studies [14].

## Conclusions

This study found 73–74% pass-through of Oakland’s SSB tax in supermarkets, grocery stores, and pharmacies, but no overall pass-through in convenience stores two years post-tax implementation. An increase in price for untaxed beverages overall was found in pharmacies but not other store types. Changes in prices varied not only across store types but also within store types by beverage type and size. For individual-size soda specifically, the price of both taxed and untaxed individual-size soda increased by 0.79–2.39 cents/ounce in convenience stores and pharmacies, whereas only taxed and not untaxed individual-size soda increased in price in supermarkets and grocery stores, by 1.05 cents/ounce. The differences by store type two years post-tax implementation highlight the heterogeneity in how different store types may be responding to the tax. These differences may lead to differential tax impacts on purchasing/consumption given disparities in access to different store types (e.g., supermarkets) by income and race/ethnicity. The results of the current study emphasize the importance of future research examining differences by store type in different taxing jurisdictions.

## Supporting information

S1 Table Changes in beverage prices in Sacramento, California, 2017–2019, from DID models for impact of the Oakland, California, sugar-sweetened beverage tax. (DOCX)

S2 Table Analytical dataset description. (DOCX)

S1 Dataset Analytical dataset. (CSV)
