# Supplementary material for: Pass-through of the Oakland, California, sugar-sweetened beverage tax in food stores two years post-implementation: A difference-in-differences study
Source: PLoS One. 2021 Jan 4;16(1):e0244884. doi: 10.1371/journal.pone.0244884 (PMC7781485; doi:10.1371/journal.pone.0244884)
Supplement: S1 Table — (DOCX) [file pone.0244884.s001.docx]

**S1 Table. Changes in Beverage Prices in Sacramento, California, 2017-2019, from DID Models for Impact of the Oakland, California, Sugar-Sweetened Beverage Tax.**

|  | **Supermarkets and Grocery Stores** | **Convenience Stores** | **Pharmacies** |
| --- | --- | --- | --- |
|  | **Coef. (95% CI)** | **Coef. (95% CI)** | **Coef. (95% CI)** |
| Taxed beverages (*n*=2516, 2172, 1142) | 0.17 (0.06, 0.29) | 0.79 (0.53,1.05) | 0.32 (0.10,0.54) |
| Soda (*n*=1104, 960, 544) | 0.19 (0.04, 0.34) | 0.39 (0.23,0.54) | 0.42 (0.19,0.64) |
| Individual-size (*n*=356, 658,142) | 0.57 (0.36, 0.78) | 0.63 (0.28,0.98) | 0.44 (-0.75,1.62) |
| Untaxed beverages (*n*=2466, 1518, 1162) | 0.22 (0.12, 0.32) | 0.49 (0.28,0.71) | 0.19 (-0.10,0.47) |
| Soda (*n*=760, 504, 440) | 0.10 (-0.06, 0.27) | 0.54 (0.28,0.79) | 0.43 (0.22,0.64) |
| Individual-size (*n*=232, 420, 130) | 0.50 (0.17, 0.82) | 0.67 (0.24,1.09) | 0.47 (-0.84,1.77) |

Estimates are from DID models comparing pre-post changes in prices in cents per ounce in Oakland, California, to those in Sacramento, California, computed as linear regression models with store and product fixed effects, with robust standard errors clustered on store. Models were weighted based on the distribution of volume sold by beverage sweetener status (sugar-sweetened, artificially sweetened, or unsweetened), type, and size in Oakland, Sacramento, and 2-mile border areas around both sites in June 2016 to May 2017, computed by the authors from Nielsen scanner data. Sample sizes by store type are shown in parentheses for each row.
Abbreviations: DID, difference-in-differences.
