# Supplementary material for: Pass-through of the Oakland, California, sugar-sweetened beverage tax in food stores two years post-implementation: A difference-in-differences study
Source: PLoS One. 2021 Jan 4;16(1):e0244884. doi: 10.1371/journal.pone.0244884 (PMC7781485; doi:10.1371/journal.pone.0244884)
Supplement: S2 Table — (DOCX) [file pone.0244884.s002.docx]

**S2 Table. Analytical Dataset Description.**

| **Variable** | **Description** |
| --- | --- |
| site | Site (Oakland or Sacramento) |
| fu | Time point (baseline or two-year follow-up) |
| busid | Store identifier |
| a1 | Store type |
| productname | Beverage name |
| oak_taxed | Indicator for whether beverage was subject to Oakland tax |
| bev_cat_7 | Beverage type |
| size_indvdl | Beverage size |
| actual_peroz | Analytical price measure |
